# Hemorrhagic Transformation in Ischemic Stroke and the Role of Inflammation

**DOI:** 10.3389/fneur.2021.661955

**Published:** 2021-05-14

**Authors:** Elena Spronk, Gina Sykes, Sarina Falcione, Danielle Munsterman, Twinkle Joy, Joseph Kamtchum-Tatuene, Glen C. Jickling

**Affiliations:** ^1^Division of Neurology, Department of Medicine, Faculty of Medicine and Dentistry, University of Alberta, Edmonton, AB, Canada; ^2^Neuroscience and Mental Health Institute, Faculty of Medicine and Dentistry, University of Alberta, Edmonton, AB, Canada

**Keywords:** ischemic stroke, hemorrhagic transformation, inflammation, hypertension, diabetes, aging, reperfusion therapy

## Abstract

Hemorrhagic transformation (HT) is a common complication in patients with acute ischemic stroke. It occurs when peripheral blood extravasates across a disrupted blood brain barrier (BBB) into the brain following ischemic stroke. Preventing HT is important as it worsens stroke outcome and increases mortality. Factors associated with increased risk of HT include stroke severity, reperfusion therapy (thrombolysis and thrombectomy), hypertension, hyperglycemia, and age. Inflammation and the immune system are important contributors to BBB disruption and HT and are associated with many of the risk factors for HT. In this review, we present the relationship of inflammation and immune activation to HT in the context of reperfusion therapy, hypertension, hyperglycemia, and age. Differences in inflammatory pathways relating to HT are discussed. The role of inflammation to stratify the risk of HT and therapies targeting the immune system to reduce the risk of HT are presented.

## Introduction

Hemorrhagic transformation (HT) is a common complication of ischemic stroke that is often exacerbated by reperfusion with alteplase (recombinant tissue plasminogen activator) or endovascular therapy (EVT) (1). It occurs when the blood-brain barrier (BBB) is sufficiently disrupted to permit extravasation of peripheral blood into the brain. When HT occurs, it increases stroke morbidity and mortality and thus is important to prevent (2).

HT can be identified in 3–40% of patients with ischemic stroke, depending on the definition used and the characteristics of the cohort studied. HT is classified using both clinical and radiological criteria as summarized in Table 1. Clinical classification distinguishes symptomatic intracranial hemorrhage (sICH) from asymptomatic intracranial hemorrhage (aICH). sICH is defined as a worsening of the National Institutes of Health Stroke Scale (NIHSS) by ≥4 points within the first 36 h of stroke onset that is attributable to HT. A limitation of the clinical classification is that HT may be missed if it does not result in a major worsening of the neurological status. This may be the case when reperfusion therapy improves the NIHSS and offsets potential worsening caused by HT. The radiological classification of HT arose from the European Cooperative Acute Stroke Study (ECASS) and distinguishes small petechial hemorrhagic infarction (HI1), confluent petechial hemorrhagic infarction (HI2), small parenchymal hemorrhage (PH1) (<30% of infarct, mild mass effect), and large parenchymal hemorrhage (PH2, >30% of infarct, marked mass effect) (3). The ECASS definition has fairly poor intra- and inter-rater agreement for all types/categories except large PH2 (4). A Heidelberg Bleeding Classification scale has been proposed to address some of the challenges and limitations of the ECASS classification.

**Table 1:** Classification systems of HT in ischemic stroke.

**HT classification system**	**Type of HT**	**Criteria**
Clinical	Symptomatic ICH (sICH)	Increase in the NIHSS by >4 points within the first 36 h of stroke onset
	Asymptomatic ICH (aICH)	Increase in the NIHSS by ≤ 4 points within the first 36 h of stroke onset
ECASS	HI1	Small petechial hemorrhagic infarction
	HI2	Confluent petechial hemorrhagic infarction
	PH1	Small parenchymal hemorrhage (<30% of infarct, mild mass effect)
	PH2	Large parenchymal hemorrhage (>30% of infarct, marked mass effect)
Heidelberg Bleeding Classification	1a HI1	Scattered small petechiae, no mass effect
	1b HI2	Confluent petechiae, no mass effect
	1c PH1	Hematoma within infarcted tissue, occupying <30%, no substantive mass effect
	2 PH2	Hematoma occupying 30% or more of the infarcted tissue, with obvious mass effect
	3	Intracerebral hemorrhage outside the infarcted brain tissue or intracranial-extracerebral hemorrhage
	3a	Parenchymal hematoma remote from infarcted brain tissue
	3b	Intraventricular hemorrhage
	3c	Subarachnoid hemorrhage
	3d	Subdural hemorrhage

**Figure 1:**
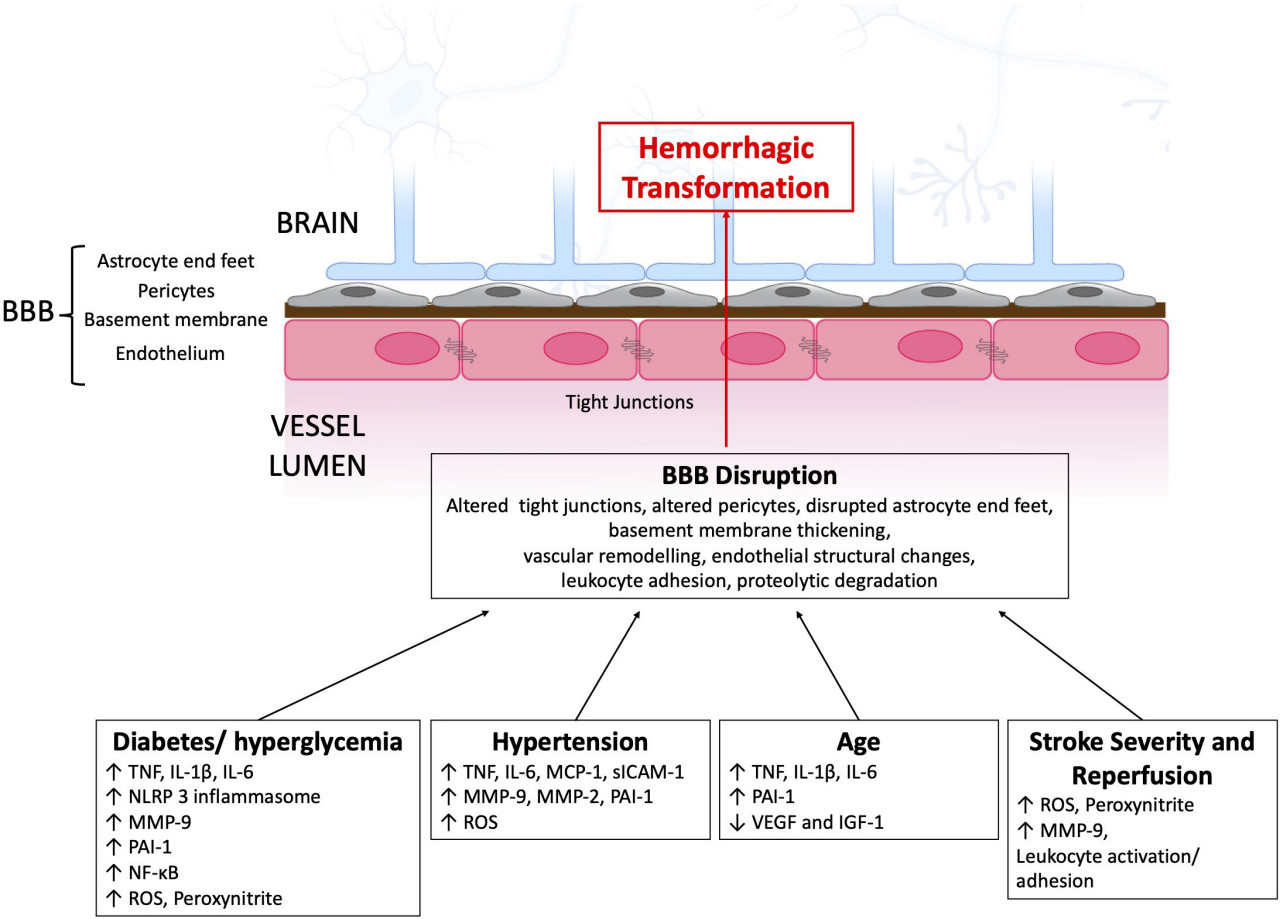
Immune pathways contributing to BBB disruption and subsequent HT after AIS. BBB, blood-brain barrier; IGF-1, insulin-like growth factor 1IL, interleukin; MCP-1, monocyte chemoattractant protein 1; MMP, matrix metalloproteinase; NF-κB, nuclear factor-κB; NLRP3, NOD-, LRR- and pyrin domain-containing protein 3; PAI-1, plasminogen activator inhibitor-1; ROS, reactive oxygen species; TLR, toll like receptor; TNF, tumor necrosis factor; VEGF, vascular endothelial growth factor.

A challenge with clinical and radiological classifications of HT is that they lack specificity to the underlying mechanism of BBB disruption and HT. Details of the mechanism of BBB disruption and the cause of HT might be included in future classification systems as our understanding of the processes involved in HT evolves. Figure 1 provides a schematic of the discussed immune pathways contributing to HT in ischemic stroke.

## Inflammation and HT

Inflammation and immune system activation are important contributors to BBB disruption and HT (5). In acute stroke activated neutrophils and monocytes produce reactive oxygen species (ROS) and matrix metalloproteinases (MMP-9, MMP-2) that contribute to BBB disruption and HT (1). Microglia, astrocytes, and endothelial cells also contribute to BBB disruption and HT through the production of MMPs, proteases, vascular remodeling, and neuroinflammation (Table 2). In the following sections, we discuss how the immune system contributes to HT specifically in the context of clinical factors related to HT, notably stroke severity, reperfusion, hypertension, diabetes, and age.

**Table 2:** Inflammatory markers associated with HT.

**Early HT (first 18 h of stroke onset)**		
**Inflammatory marker**	**Source/mechanism**	**Role in HT**
ROS (increase)	Intracellular mitochondria, nicotinamide adenine dinucleotide phosphate (NADPH)-oxidase xanthine oxidases, cellular membrane receptors inflammatory mediators, astrocytes	Disrupt the NVU (endothelial-pericyte-astrocyte) leading to increase BBB degradation
HMGB1	Microglia, astrocytes	Upregulates MMP-9 via TLR4. TNF, IL-1β
Peroxynitrite	Micro vessels, neurons and astrocytic end feet	Activate MMPs, disrupting vascular integrity
NF-κB	Astrocytes, microglia, and endothelial cells	Part of neutrophil infiltration pathway, upregulate cell adhesion molecules and inflammatory cytokines.
Leukocytes (increase)	Circulating leukocytes adhere to vascular endothelial cells following ischemia. Leukocyte adhesion and migration across the vasculature activates a number of signaling cascades (protein kinase C, focal adhesion kinase) that increase BBB permeability	Signaling cascade increased BBB permeability through ROS and MMP-9 expression.
MMP-9 (blood derived) (increase)	Leukocytes (Neutrophils) (not primary source). Mechanisms for MMP-9 activation following ischemia include: (1) ROS (2) TNF, IL-1β, and other cytokines that activate MMP-3 which converts proMMP-9 to active MMP-9 (3) actions of high mobility group box protein1 (HMGB1) on TLR4 receptors that then induce MMP-9 or (4) NF-κB induction of MMP-9	Luminal side: acts on TJP (tight junction proteins) (claudin-5, occludin, ZO-1) and basal lamina proteins (fibronectin, lamina, collagen), taken into endothelial cells or acts on basal lamina to open the BBB. Inside brain: Neutrophils can release MMPs that act directly on TJPs and/or basal lamina to disrupt the NVU (endothelial-pericyte-astrocyte)
MMP-2 (brain derived) (increase) remains elevated for days post-stroke	Astrocytes, endothelial cells and leukocytes	MMP-2 mediates degradation of occludin (tight junction protein)
Leukocyte gene expression	Six genes identified through mRNA expression: amphiregulin (AREG), membrane-associated ring finger (C3HC4) 7 (MARCH7), SMAD family member 4 (SMAD4), inositol polyphosphate-5-phosphatase (INPP5D), multiple coagulation factor deficiency 2 (MCFD2) and vascular endothelial growth inhibitor (VEGI)	
**Late HT (18–24 h of stroke onset)**		
	**Source**	**Role**
MMP-9 (Brain derived) (Increase)	MMP-9: astrocytes, neurons, microglia and endothelial cells. Activated by from ROS, TNF and IL-1β, HMGB1, NF-κB induction	Disruption of BBB
MMP-3 (Brain derived) (Increase)	MMP-3: pericytes and endothelial cells. MMP-3 acts on proMMP-9 to produce active MMP-9 and thus may promote HT	Disruption of BBB
Role of Vascular Remodeling	• A number of growth factors, MMPs and other molecules form new vessels and NVU • VEGF plays an important role in vascular remodeling and angiogenesis	VEGF: early, promotes BBB disruption; later promotes BBB integrity and vascular function. MMPs: Promote new vessel formation and increased pericyte/endothelial expression of tight junction proteins (ZO-1, occludin, claudin-5) HMGB1: acts on endothelial progenitor cells to promote peri-infarct angiogenesis (beneficial role)
ROS		Act as signaling molecules to regulate cell growth, differentiation and angiogenesis.

*BBB, blood-brain barrier; HMGB1, high mobility group box protein 1; IL, interleukin; MMP, matrix metalloproteinase; NF-κB, nuclear factor-κB; NVU, neurovascular unit; ROS, reactive oxygen species; TLR, toll like receptor; TNF, tumor necrosis factor; TJP, tight junction protein; VEGF, vascular endothelial growth factor*.

Several trials have focused on improving stroke outcomes through targeting immune factors. There has yet to be immunomodulator identified to reduce the risk of HT. A summary of discussed treatments and their molecular targets is provided in Table 3.

**Table 3:** Therapeutic targets in acute ischemic stroke focused on modulating HT.

**Therapy**	**Target**	**Effect on ischemic stroke**	**References**
3K3A-APC (modified active protein C)	Protease-activated receptor 1	Possible benefit of reduced and smaller hemorrhages.	(6)
Enlimomab	ICAM-1	Associated with poor outcomes and increased HT	(7)
Edaravone	ROS	No benefit in reducing HT occurrence	(8)
Glycyrrhizin	HMGB1	• In rat model decreased HT • No human trial yet	(9)
Minocycline	MMP-9	No benefit	(10)
N-tert-butyl-α-phenylnitrone (PBN)	ROS	• In rat and rabbit models helped to decrease HT • No human trial yet	(11, 12)
NXY-059	ROS	Found to be ineffective for preventing HT	(13)
Otaplimastat	MMP	Further investigation required	(14)

*HMGB1, high mobility group box protein 1; ICAM-1, intercellular adhesion molecule-1; MMP, matrix metalloproteinase; ROS, reactive oxygen species*.

## Clinical Risk Factors for HT

Clinical features associated with an increased risk of HT in patients with ischemic stroke include advanced age, stroke severity (NIHSS), hypertension, hyperglycemia, poor collaterals, early infarction on brain imaging, low platelet count, use of antithrombotic drugs, and reperfusion therapy (15). Many of these factors are associated with inflammation and activation of the immune system, as discussed below. The clinical features have been summarized into scores to estimate the risk of HT and guide reperfusion therapy (Table 4). However, clinical characteristics and risk scores fail to identify all patients who experience HT, highlighting that a gap remains in our understanding of the pathophysiology of HT. Variables most commonly included in HT risk scores are stroke severity (admission NIHSS), hyperglycemia, hypertension, and age.

**Table 4:** Risk scores proposed for HT prediction with alteplase administration.

**Score**	**Name of score**	**Components**	**References**
HTI – 0 to 6 points	Hemorrhagic transformation index score	ASPECTS (Alberta Stroke Program Early CT score), NIHSS, hyperdense middle cerebral artery sign, and presence of atrial fibrillation on ECG at admission	(16)
GRASPS	Glucose, Race, Age, Sex, Systolic blood Pressure, Severity	Glucose at presentation, race [Asian], age, sex [male], systolic blood pressure at presentation, and severity of stroke at presentation [NIH Stroke Scale]	(17)
HAT	Hemorrhage after thrombolysis	NIHSS score, hypodensity on CT scan (initial), serum glucose at baseline, and history of diabetes	(18)
HeRS	Hemorrhage Risk Stratification	Age, infarct volume, eGFR	(19)
HeRS plus	Hemorrhage Risk Stratification Plus	Addition of serum glucose, WBC count, and warfarin use on admission	(19)
SITS-sICH	Safe Implementation of Treatment in Stroke – Symptomatic IntraCerebral Hemorrhage risk	NIHSS score, serum glucose, systolic blood pressure, age, body weight, stroke onset to treatment time, aspirin or combined aspirin and clopidogrel, and history of hypertension	(20)
SEDAN Score	Hemorrhage risk after thrombolysis	Blood Sugar, Early infarct sign, Dense artery signs, Age, and NIHSS score	(21)

*NIHSS, National Institutes of Health Stroke Scale*.

## Hemorrhagic Transformation and Stroke Severity

There is a strong relationship between duration and severity of brain ischemia and the risk of HT in both patients with stroke and experimental stroke models. Increased time from stroke onset is associated with larger core volumes, a higher degree of vascular disruption, and therefore a higher risk of HT. Increased time from ischemia onset to reperfusion has been associated with an increased risk of HT whether they received thrombolytic therapy or not (1). Recanalization beyond 6 h of stroke onset is an independent predictor of HT in human stroke, while early reperfusion is associated with a reduced risk of HT. Increased NIHSS is also associated with risk of HT. Patients with an NIHSS < 10 had <13% rate of HT. In comparison, patients with NIHSS > 15 had >50% rate of HT (22). Activation of the immune system is related to severity of infarction. Whether the greater immune activation from a larger infarct increases risk of HT remains unclear and warrants further evaluation.

## Thrombolytic Therapy and HT

Treatment with alteplase is associated with a 6–8% risk of sICH (23–27). This is mediated in part by the fact that thrombolytics breakdown blood clots and recanalize occluded cerebral vasculature. Recanalization of an occluded blood vessel can promote BBB disruption, contributing to reperfusion injury and increased risk of HT (1, 28). Activation of platelets, coagulation factors, and the innate and adaptive immune systems also contribute to injury following restoration of blood flow (29, 30).

Alteplase can also promote HT through non-fibrinolytic mechanisms, including activation of the immune system (31). Alteplase promotes neutrophil degranulation and release of MMP-9 (32). It acts on protease-activated receptor 1 (PAR-1) to increase MMP-9 expression and disrupt the BBB (33). Additionally, alteplase activates platelet-derived growth factor-CC (PDGF-CC), an agonist of platelet-derived growth factor receptor alpha (PDGFRa) on astrocyte end-feet (34). This can promote BBB disruption and HT, as PDGFRa activates MMPs (33). Alteplase also binds the lipoprotein receptor (LRP) on neurons and perivascular astrocytes to induce MMP-3 and MMP-6 expression (33). While inhibition of MMPs can reduce the risk of HT in animal stroke models, evidence in humans is lacking. Minocycline inhibits MMP-9 and has been evaluated in the MINOS trial (10). A phase 4 trial was later terminated due to lack of effect. MMP-9 inhibition, while potentially beneficial to reduce the risk of HT, may also impair post-stroke vascular remodeling and impair recovery. Indeed, inhibition of MMP-9 at 24–48 h after stroke worsens outcome in animal models (35). A phase 2 study, SAFE-TPA, evaluated different doses of Otaplimastat, a neuroprotectant inhibiting the MMP pathway (14). In animal models, Otaplimastat in combination with alteplase helped to reduce edema and HT. While Kim et al. (14) found the drug to be safe in patients, further investigation is required to determine effect in stroke.

Evidence is emerging regarding the use of Tenecteplase (TNK) in acute ischemic stroke (36, 37), a current treatment for acute myocardial infarction. TNK promotes the conversion of plasminogen to plasmin which degrades fibrin-based clots. It is a variant of recombinant tPA (tissue plasminogen activator), with higher resistance to plasminogen activator inhibitor-1 (PAI-1) and enhanced fibrin specificity (38). Studies of TNK in stroke are ongoing. In the EXTEND-IA TNK Trial, recanalization rates before thrombectomy were higher in the TNK group (22%, *n* = 101) compared with the alteplase group (10%, *n* = 101) with improved functional outcomes and similar risk of HT (1% sICH and 5–6% HT PH2) (37). As with alteplase, TNK may promote HT through several mechanisms that warrant further investigation.

Experimental evidence supports a role of reactive oxygen species (ROS) and reactive nitrogen species (RNS) in early HT. Superoxide and peroxynitrite can disrupt microvascular integrity and may contribute to HT (1). Cerbral ischemia and reperfusion produce reactive oxygen and nitrogen species including superoxide, nitric oxide (NO), and peroxynitrite (ONOO^−^) in microvessels, neurons and astrocyte end feet (39). Peroxynitrite can activate MMPs further disrupting vascular integrity. Hyperglycemia, as described in the section Hyperglycemia and HT can also enhance ROS and peroxynitrite.

High-mobility group box 1 (HMGB1) is released from injured cells of the central nervous system such as microglia and astrocytes (40). HMGB1 binds to its receptors (RAGE, toll-like receptor 2 (TLR2), and TLR4) and augments inflammation via the upregulation of tumor necrosis factor (TNF), interleukin 1β (IL-1β), and other cytokines (41). HMGB1 upregulates MMP-9 in neurons and astrocytes via TLR4. This could be a proposed target to help dampen the inflammatory response in cerebral ischemia and prevent HT as discussed in the section Hyperglycemia and HT.

Nuclear factor-κB (NF-κB) acts as a complex regulator of the innate immune system leading to activation of cellular adhesion molecules such as ICAM-1, VCAM-1 and E-selectin and inflammatory cytokines, mediating the neutrophil infiltration pathway (42, 43). A study targeting HIF-1, hypoxia-inducible factor 1, a transcription factor that works to maintain homeostasis under hypoxia, found that BBB integrity was protected through suppressing the HMGB1/TLR4/NF-κB pathway to reduce the risk of alteplase-induced HT (44).

In experimental studies, Enlimomab, an ICAM-1 antibody, reduced leukocyte adhesion and infarct size (7). In the Enlimomab Acute Stroke Trial, it was associated with poor outcomes and increased HT.

In the RHAPSODY phase 2 trial, 3K3A-APC (a modified APC), a recombinant variant of human activated protein C (part of the coagulation pathway), was evaluated as a neuroprotectant in acute ischemic stroke treated with alteplase and or mechanical thrombectomy (6). 3K4A-APC acts on the protease-activated receptor 1 (PAR1) to promote vascular integrity and reduce neurological injury. The study showed a trend toward reduced HT, but further confirmation is required. However, targeting the PAR1 receptor shows promise as a strategy to reduce HT in stroke patients treated with reperfusion therapy.

## Endovascular Therapy and HT

Endovascular thrombectomy is of benefit in patients with acute large vessel occlusion and salvageable brain tissue (45). In a metanalysis of SWIFT PRIME, ESCAPE, EXTEND-IA, and REVASCAT the risk of sICH was 2.5% in EVT treated compared to 2.8% in non-EVT treated patients (*p* = 0.76), and the risk of PH was 8% in both EVT-treated patients and non-EVT patients (*p* = 0.96) (46). In the HERMES pooled analysis of MR CLEAN, ESCAPE, REVASCAT, SWIFT PRIME, and EXTEND IA stroke trials, the rate of sICH was 4.4% in the EVT group compared to 4.3% in the medical therapy group (45). Thus, the rate of HT is not significantly higher in the patients enrolled in EVT trials compared to controls receiving alteplase alone.

The DIRECT-MT trial compared EVT-only to EVT plus alteplase (47). The rates of HT were comparable between groups, with sICH occurring 4.3% in EVT group vs. 6.1% in EVT + alteplase (*p* = 0.30) and aICH occurring in 33.3% of the EVT group 36.2% in the EVT + alteplase group (p0.45). Similar rates of HT in EVT + alteplase vs. EVT alone have been reported by others (45, 48).

Thus, reperfusion with EVT may have lower rate of sICH than might be expected. EVT can provide a more rapid recanalization of large vessel occlusions which may reduce severity of ischemic brain injury, and thus reduce risk of HT. This is particularly true when EVT-treated patients are selected for small core and large penumbra by imaging. Core size does relate to risk of HT, with HT being higher in EVT-treated patients with larger core on baseline imaging (49). EVT may also produce differences in the type BBB disruption compared to alteplase. In an animal model, alteplase produced a more diffuse pattern of BBB disruption, whereas EVT had a more concentrated BBB disruption in central areas of ischemia (50). Additional evaluation is needed to understand HT in EVT-treated patients including differences in BBB disruption and potential benefits and risks of rt-PA (alteplase) use before EVT.

## Hyperglycemia and HT

Hyperglycemia is present in approximately 28–40% of patients with acute ischemic stroke (51–53). It can relate to pre-existing diabetes mellitus (DM) or a stress response leading to a rise in cortisol, glucose and catecholamines. For both diabetic and non-diabetic patients, hyperglycemia increases the risk of HT and is associated with worse clinical outcomes (51). There are conflicting reports concerning infarct growth in a hyperglycemic setting relative to normal glycemic levels (54–56). For the most part, hyperglycemia is associated with more rapid infarct growth and worse outcomes. In a review of 426 patients, higher values of HbA1c were associated with a higher risk of HT in patients with fasting blood glucose > 7.8 mmol/L (27% higher risk) and in those with fasting blood glucose <7.8 mmol/L (36% higher risk) (57). Higher levels of HbA1c were also associated with a 48% higher risk of poor functional outcome. Treatment of hyperglycemia is recommended by stroke guidelines for the management of patients with acute ischemic stroke (58). Hyperglycemia is a risk factor for HT in both alteplase-treated patients and those not receiving thrombolysis. This association might be driven by hyperglycemia's effects on brain vasculature and the inflammatory response.

Hyperglycemia can potentiate inflammation resulting in increased BBB disruption and HT (59). Factors related to hyperglycemia that can increase the risk of HT are presented in Table 5. Diabetes is associated with an increase in plasma TNF, IL-1β, interleukin 6 (IL-6), interferon-g (IFNγ), and PAI-1, as well as alteration in immune cell response leading to chronic low-grade inflammation (60). An increase in PAI-1 may decrease the efficacy of alteplase and increase infarct size (67). This may result in increased risk of HT (68, 69). Hyperglycemia also activates nuclear factor-κB (NF-κB) in astrocytes, and enhances leukocyte adhesion through higher expression of cell adhesion molecules in cerebral blood vessels (ICAM1, intracellular adhesion molecule 1; VCAM-1, vascular cell adhesion molecule 1 and E-selectin) and the production of pro-inflammatory cytokines (TNF, IL-6, and IL-1β) (42, 70).

**Table 5:** Summary of factors in hyperglycemia that promote BBB disruption and risk of HT.

**Marker**	**Effect on BBB/consequences/contribution to risk of HT**	**References (for further reading)**
**Inflammation associated with diabetes**		
Increase in chronic inflammatory cytokines: TNF, IL-1β, IL-6, and PAI-1	Impacting inflammation response (BBB permeability) PAI-1 interferes with tPa/alteplase degradation	(60)
NLRP3 Inflammasome	Associated with chronic inflammation in T2DM	(61)
**Inflammation associated with hyperglycemia and ischemia**		
Activation of NF-κB. Inflammatory response: includes TNF, IL-1β, IL- 6, sICAM-1, ICAM-1, VCAM-1 and E-selectin activation. Source: astrocytes	Inflammatory cascade attracting leukocytes to the ischemic area.	(42)
Increase in MMP-9 (associated with ischemia)	Disruption of BBB	(54, 55)
Increased adhesion of neutrophils	NVU disruption	(62)
Increased superoxide production, NADPH	Disruption of BBB	(63)
Peroxynitrite	• HMGB1 leading to activating leukocytes and inflammatory cytokines. • Activation of NLRP3 leading to neutrophil recruitment and BBB disruption	(9) (64)
**Mechanical cellular changes due to chronic hyperglycemia**		
Capillary basement membrane thickening and enhanced microvascular permeability	Diabetes complications impacting the BBB structure	(60)
Decrease in pericytes	Diabetes complications impacting the BBB structure (NVU)	(65)
Decreased tight junction proteins (occludin and claudin-5)	Disruption of BBB	(66)

*BBB, blood-brain barrier; E-selectin, endothelial-leukocyte adhesion molecule 1; HMGB1, high mobility group box protein 1; ICAM-1, intercellular adhesion molecule-1; IL, interleukin; MMP, matrix metalloproteinase; NF-κB, nuclear factor-κB; NLRP3, NOD-, LRR- and pyrin domain-containing protein 3; NVU, neurovascular unit; PAI-1, plasminogen activator inhibitor-1; sICAM, soluble intercellular adhesion molecule; T2DM, type 2 diabetes; TNF, tumor necrosis factor; tPa, tissue plasminogen activator; VCAM-1, vascular cell adhesion molecule 1*.

Neutrophils from patients with diabetes have enhanced capacity for endothelial adhesion (62). In stroke models using diabetic rats, hyperglycemia increases neutrophil adhesion and transmigration, and also increases expression of TNF, IL-1β, E-selectin, soluble intercellular adhesion molecules (sICAMs), endothelial NO synthase (eNOS) and inducible nitric oxide synthase (iNOS) (54, 71). In a rat model, hyperglycemia increases infarct volume, worsens outcome and increases risk of HT (55). Additionally, hyperglycemia increased the activity of MMP-9. Risk of HT is increased by glucose injection regardless of the time of ischemia (54, 55). Hyperglycemia can also increase superoxide production through NADPH oxidase, resulting in greater BBB disruption and HT. Apocynin, an NADPH oxidase inhibitor, prevents BBB disruption and HT in animal models of stroke (63).

In a rat model of stroke, hyperglycemia enhanced release of HMGB1 and the rate of alteplase related HT (9, 40, 56). Treatment with glycyrrhizin, a direct HMBG1 inhibitor, downregulated the expression of NADPH. This decreased superoxide and peroxynitrite(ONOO^−^), reduced TLR2, and MMP-9, and preservation of type IV collagen and claudin-5, thus attenuating HT and BBB damage. The role of an HMGB1 inhibitor in patients with stroke remains to be determined.

NLRP3 (NOD-like receptor protein 3) inflammasome can also promote BBB disruption and HT. In a MCAO rat model with acute hyperglycemia and increased expression of NADPH oxidase, iNOS induction lead to the production of superoxide, nitric oxide and peroxynitrite (56). It was suggested that peroxynitrite could activate NLRP3 inflammasome in hyperglycemia, a mediator in HT following reperfusion. Inhibiting the NLRP3 inflammasome minimized HT *in vivo*, and could potentially offer a treatment option.

NLRP3 inflammasome is also involved in chronic inflammation associated with type 2 diabetes (T2DM) (61). Primary activation of NLRP3 leads to transcription of pro-IL-1β and pro-IL-18 into mature IL-1β and IL-18. Active IL-1β is then responsible for increased expression of adhesion molecules, leading to neutrophil recruitment and contributing to BBB disruption (64). Inhibiting NLRP3 may reduce the impact of stroke in patients with diabetes by regulating neutrophil recruitment. Hyperglycemia may upregulate TXNIP-NLRP3 inflammasome causing alteplase induced BBB disruption and HT (72). Thioredoxin interacting protein (TXNIP) mediates hyperglycemia-induced oxidative damage and inflammation in the brain, while reducing cerebral glucose uptake/utilization.

Other mechanisms by which hyperglycemia can promote BBB disruption and cause HT include a decrease in tight junction proteins occludin and claudin-5 (66), chronic oxidative stress and thickening of microvascular basement membrane, increase of microvascular permeability (60), and loss of pericytes (65).

### Effect of Hyperglycemia on Treatment With Alteplase

Hyperglycemia impairs the fibrinolytic activity of alteplase, particularly in the acute phase of treatment, which may result in larger infarct size with more severe ischemia and thus, higher risk of HT (63, 73–75). Type 2 DM is associated with increased blood levels of PAI-1, an inhibitor of tPA (endogenous and exogenous) (76). Hyperglycemia also affects the coagulation system by increasing thrombin production and stimulating the intrinsic tissue factor pathway, thus affecting reperfusion and contributing to HT (77). In rats with Type 1 DM, treatment of acute stroke with alteplase increased MMP-9 activity, BBB breakdown, brain hemorrhage, and worsened functional outcomes (75).

### Treatment of Hyperglycemia to Prevent HT

Several stroke trials have evaluated the benefit of treating hyperglycemia to reduce the risk of HT. Control of hyperglycemia through insulin infusion was tested in patients in the UK Glucose Insulin in Stroke Trial (GIST UK) (78) and the SHINE trial (51). In the SHINE trial, 80% of the patients had Type 2 DM. In the intensive treatment group, the average blood glucose was 6.6 mmol/L compared to 9.9 mmol/L in the standard treatment group. No net improvement in stroke outcome was identified, despite a significant reduction in blood glucose achieved with insulin infusion. Potential acute lowering of glucose does not fully mitigate downstream effects already initiated by hyperglycemia such as enhanced inflammation, endothelial cell dysfunction, neutrophil adhesion, MMP-9 activity, and ROS production. There is an ongoing secondary analysis of the effect of acute insulin therapy on the risk of HT, which may provide further insights into the link between hyperglycemia and HT from the SHINE trial.

A systematic review of 274 research articles aimed to identify the ideal target and strategy for blood glucose management (79). Glucose control was recommended to start within the first few hours of stroke onset. Tight glucose control was not recommended due to the risk of hypoglycemic events. Glycemic variability is to be avoided, as it is associated with adverse outcomes.

## Hypertension and HT

An elevated blood pressure can increase the risk of HT through a variety of mechanisms, including direct mechanical effects on the cerebral/brain vasculature, exacerbated inflammation, and vascular remodeling affecting collateral circulation, endothelial function, and autoregulation (80). A summary of factors in hypertension that impacted HT through BBB modification is shown in Table 6.

**Table 6:** Summary of factors related to hypertension that promote BBB disruption and risk of HT.

**Marker**	**Effect on BBB/consequences/contribution to risk of HT**	**References (for further reading)**
**Changes to the immune system and response**		
Elevated levels of proinflammatory cytokines as TNF, IL-6, MCP-1, and sICAM-1 in the vasculature	Impact endothelial cell ability to regulate. Leukocyte interaction and infiltration, disruption of BBB	(80)
**Response to Angiotensin II due to chronic hypertension**		
Increased MMP-9 and MMP-2	Activated by the renin-angiotensin system in the vasculature, leading to vessel remodeling and disruption of the BBB	(81)
Increase in serum PAI-1	Activated by Angiotensin II, interferes with the tPa/alteplase degradation	(67)
Increased ROS through NADPH oxidases	Macrophages in response to Angiotensin II Additionally, acute hypertension may attenuate the ability to cope with ROS	(82)
**Mechanical cellular changes due to chronic hypertension**		
Remodeling of microvasculature: Decreased lumen diameter, increased vascular resistance, increased wall to lumen diameter.	Decreased endothelial cell function and reduced autoregulation ability	(80)

*BBB, Blood brain barrier; IL, interleukin; MCP-1, monocyte chemoattractant protein 1; NADPH, Nicotinamide adenine dinucleotide phosphate; PAI-1, plasminogen activator inhibitor-1; ROS, reactive oxygen species; sICAM, soluble intercellular adhesion molecule; TNF, tumor necrosis factor; tPa, tissue plasminogen activator*.

Hypertension is common in patients with ischemic stroke and associated with increased risk of HT (20). Blood pressure is elevated in over 60% of patients with acute stroke (83). The elevated blood pressure may be caused by several components, including pre-existing or untreated hypertension, stress response (transient response), increased intracranial pressure, disrupted central autonomic regulation, activation of the neuroendocrine system, or inflammatory responses.

### Impact of Hypertension on Inflammation and BBB Permeability

Hypertension is associated with disruption of the BBB which could increase risk of HT (84). In the spontaneous hypertensive rat, BBB disruption can be observed as early as 3 months of age (85). The renin-angiotensin system is involved in hypertension and contributes to BBB disruption. It activates MMPs, causing an increase in circulating and tissular MMP-2 and MMP-9 (81, 86). The increase of angiotensin II in hypertension induces perivascular macrophages to produce ROS and contribute to BBB disruption (82, 84). Hypertension is associated with an increase in blood levels of pro-inflammatory cytokines such as TNF, IL-6, monocyte chemoattractant protein-1(MCP-1), and sICAM-1 which can contribute to BBB disruption (80). Hypertension is associated with a decreased expression of cerebral endothelial tight junction proteins (claudin-3, claudin-5, and claudin-12), thus enhancing BBB permeability (84). Angiotensin II causes an increase in serum PAI-1 levels, thus reducing the effectiveness of alteplase (69).

### Hypertension Related Vascular Remodeling

Chronic hypertension alters the cerebral vasculature, with microvascular rarefaction, arterial remodeling, and increased BBB permeability (80). Hypertension is associated with a decrease in lumen diameter, increase in vascular resistance, and impairment of endothelial function. These effects impair collateral circulation, reducing capacity to maintain adequate oxygenation when a cerebral artery occlusion occurs. Poor collateral circulation, which sustains the penumbra, results in faster expansion of infarct core, associated with risk of HT (87, 88).

### Impact of Hypertension on HT Following Reperfusion Therapy

In a rabbit clot model, hypertension induced before and after alteplase administration increases the risk of HT (89). The size of HT also relates to the peak mean arterial blood pressure. Decreasing pre-stroke blood pressure in rats reduced the risk of HT (90). BBB changes may be mechanical or result from acute changes related to endothelial or inflammatory activation.

Maintaining the blood pressure below 185/110 mmHg is recommended to reduce the risk of HT in patients treated with alteplase (91). The risk of HT increases with each 10 mmHg rise in systolic blood pressure from 140 to 180 mmHg (92). Observational studies suggest lower blood pressure within the first 24 h is associated with more favorable outcomes and less frequent sICH (93). In addition, the risk of HT following alteplase is higher in patients with greater blood pressure variability, particularly in the first 6 h (94), and in patients with elevated systolic blood pressure (95). An elevated systolic blood pressure (SBP) is also associated with worse outcomes and higher risks of HT in patients treated with EVT (96, 97).

### Hypertension Treatment to Prevent HT

The ENCHANTED trial compared the benefit of intensive blood pressure control (SBP target of 130–140 mmHg) to that of the standard of care (SBP <180 mmHg) (98). Intensive lowering of blood pressure was safe and decreased the incidence of HT from 18.7 to 14.8% (*p* = 0.0137). However, despite a reduction in HT, no net improvement in the 90-day Modified Rankin Scale (mRS) was observed. The best stroke outcomes are observed when BP is maintained between 140 and 180 mmHg (99). It is challenging to understand the ideal blood pressure for stroke treatment. In an acute setting, a rise in BP may be beneficial for preservation of brain perfusion in case of large vessel occlusion (opening of collateral vessels) while intensive BP lowering in the hyperacute phase (<48 h) may be detrimental in the context of impaired regulation of the cerebral circulation. The RIGHT-2 trial showed that functional outcomes were not improved with blood pressure reduction prior to stroke treatment (100). The Dutch ThRombolysis in Uncontrolled Hypertension (TRUTH) trial is an ongoing study evaluating whether actively lowering blood pressure below 185/110 mmHg affects stroke outcome in alteplase treated patients (101).

The BP-TARGET aimed to evaluate intense systolic blood pressure targets (100–129 mm Hg) and standard care systolic targets (130–185 mm Hg) for 24 h after reperfusion (102). Intensive blood pressure control was not superior to standard of care for the prevention of HT. A comment letter suggest that alternative variables should also be considered, such as collateral circulation, blood pressure variability at baseline and during monitoring (103).

## Age and Risk of HT

Advancing age is associated with an increased risk of HT (20, 104, 105), as well as worse stroke outcomes (106, 107). Infarct size is not consistent between sources; however, most authors suggest an increase in infarct size. Despite an increased risk, patients of older age derive similar benefit from thrombolysis (108, 109). The rationale for age to increase the risk of HT is multifactorial, including an increase in systemic inflammation and BBB permeability. Additionally, elderly patients are also more likely to be on antithrombotic treatment, have a greater burden of cerebrovascular disease, and are more likely to have comorbid diseases such as hypertension and diabetes. These comorbidities contribute to inflammation, notably atherosclerosis, diabetes, hypertension, and hyperlipidemia. When stroke occurs, this age-associated inflammation may contribute to BBB disruption leading to an increased risk of HT. Table 7 summarizes the mechanisms through which age affects the risk of HT.

**Table 7:** Summary of factors related to age that impact HT and BBB.

**Marker**	**Effect on BBB/consequences/contribution to risk of HT**	**References (for further reading)**
**Changes to the immune system and response**		
Inflammation: low grade chronic inflammation. Elevated levels of proinflammatory mediator's TNF, IL-1β, IL-6	Effect the inflammatory response and BBB permeability/ function	(110–112)
Increase in PAI-1	Interferes with the tPa/alteplase degradation	(113)
Decrease in VEGF and IGF-1	Alter the angiogenesis response	(114)
**Changes to the inflammatory response**		
Enhanced neutrophil response/recruitment	Increased MMP-9 and ROS	(106)
Enhanced microglial response	Enhanced inflammation and BBB permeability	(115)
**Mechanical cellular changes due to aging**		
Endothelial cells change in structure and adopt a senescence phenotype	Increased ROS which reduces NO activity	(116)
Decrease in pericytes	Mechanical disruption to BBB permeability	(105)
Astrocytes change structure	Mechanical disruption to BBB permeability	(105, 115)

*BBB, Blood brain barrier; IGF-1, insulin-like growth factor 1; IL, interleukin; NO, nitric oxide; PAI-1, plasminogen activator inhibitor-1; TNF, tumor necrosis factor; tPa, tissue plasminogen activator; VEGF, vascular endothelial growth factor*.

### Effect of Age on the Immune System

Advanced age is characterized by immunosenescence, gradual immune dysregulation and changes to cell function, leading to a reduced capacity to respond to antigen stimulation (110). Aging is also associated with inflammaging: chronic inflammation with elevated plasma concentrations of TNF, IL-1β, and IL-6 predictive of fragility, mortality, and functional disability (110–112). This chronic low-grade inflammation state alters the innate immunity of the brain and may contribute to the risk of HT.

Aging impacts both the innate and adaptive immune systems (110). Over time, the availability of hematopoietic stem cells declines, leading to decreased capacity to renew peripheral immune cells. Fewer naive B-lymphocytes are produced from the bone marrow, and T cell diversity is also reduced after age 70. Cells of the myeloid lineage are also affected by age, neutrophils have decreased phagocytic capacity and a reduced oxidative burst, leading to an increased inflammatory time period (110, 112). Moreover, there is reduced production of cytokines from macrophages which have decreased phagocytic capacity (106, 110). During stroke, young mice tend to recruit monocytes, whereas aged mice have greater recruitment of neutrophils. Additionally, higher levels of ROS and MMP-9 are associated with increased age. As discussed in the section Inflammatory Biomarkers and Prediction of HT, an increased neutrophil to leukocyte (NIL) ratio was independently associated with HT (117). Furthermore, with advancing age, microglia may become more prone to generate an inflammatory response when stroke occurs (115). With advancing age, there is a dysregulation in microglia pathways leading to a prolonged and amplified immune response, thus potentially increasing the risk of HT.

### Age and the Neurovascular Unit

The neurovascular unit also undergoes age-related changes which may contribute to the risk of HT (104, 105). Aged cerebral endothelial cells have a smaller cytoplasm and dysfunctional mitochondria that adopt a senescent phenotype, promoting low-grade inflammation. Endothelial cells also produce more ROS with age, which reduces NO activity and limits the vasodilation capacity (116). Moreover, the number of pericytes, vital for BBB integrity, decreases with age, with a negative impact on the microcirculation, reducing the ability to maintain the BBB integrity after stroke (105). Astrocytes can become more inflammatory with age, which may also contribute to the risk of HT (115). Additionally, astrocytes undergo clasmatodendrosis with age and retract their end-feet, thus altering BBB integrity (105, 115). Finally, with aging, miRNA and RNA expression for genes associated with vascular tone, tight junction protein expression and cell adhesion are downregulated, impacting the BBB.

### Age and Cerebral Vasculature

Angiogenesis is a necessary process to reduce infarct volume and support recovery after stroke. The most critical factors for supporting capillary angiogenesis include fibroblast growth factor (FGF), TNF, transforming growth factor-beta (TGF-β), and the angiopoietins (Ang) (118). With age, there is an attenuated pro-angiogenic response, decreased expression of vascular endothelial growth factor (VEGF) and insulin-like growth factor 1 (IGF-1) possibly contributing to the risk of HT (114). VEGF has an important role in vascular remodeling and angiogenesis and is associated with delayed HT and vascular remodeling (1). VEGF has a biphasic role in stroke. Early after stroke, VEGF promotes BBB disruption and HT but supportes vascular function and BBB integrity at a later stage. In rats, VEGF signal inhibition attenuates alteplase-related HT (119). Age is associated with a decrease in the number and diameter of native collaterals as well as impaired collateral remodeling in the brain (120). Collaterals influence the rate of HT after thrombolytic treatment (121). Thus, the increased risk of HT with age may also relate to an age-associated reduction in collaterals.

## Inflammatory Biomarkers and Prediction of HT

Several protein and transcription biomarkers have been evaluated to predict the risk of HT. This includes plasma cellular-fibronectin (c-Fn), a major component of extracellular matrix involved in wound healing and cell adhesion (122); MMPs (122, 123); neutrophil and lymphocyte counts (117, 123); ferritin (123), vascular adhesion molecule−1 (VAP-1), and IL-10 that are associated with alteplase induced HT (124, 125). Additionally, inflammatory markers such as TNF, C-reactive protein and homocysteine are associated with the risk and severity of HT (126). Fibrinolysis inhibitors [PAI-1, lipoprotein(a), thrombin-activated fibrinolysis inhibitor (TAFI), and homocysteine] may help to predict sICH after thrombolysis (69, 122, 124).

Ischemic stroke elicits a strong local and peripheral inflammatory response. There is an increase in inflammatory cytokines (e.g., TNF, IL-1β, IL-6), cell adhesion molecules (e.g., ICAM-1, VCAM, P-selectin), damage-associated molecular patterns [DAMPs, e.g., high mobility group box protein 1 (HMGB1), peroxiredoxin, mitochondrial DNA], MMPs and ROS (127). Microglia, cerebral endothelial cells, and peripheral leukocytes are also activated during the immune response. This results in the recruitment, adhesion, and infiltration of the ischemic brain by peripheral leukocytes. Aspects of the inflammatory response alters the structure of the BBB, reorganizing tight junction proteins and the actin cytoskeleton, and contributing to HT (128).

A few trials have targeted ROS. NXY-059 targeting ROS was trialed to reduced alteplase-related intracranial hemorrhages (13). In animal studies, NXY-059 was associated with improved functional recovery and reduced infarction size. In the human trial, NXY-059 was found to be ineffective for preventing HT. Additionally, as part of a retrospective study in Japan, Edaravone administration with alteplase (rtPA) found that there may be a benefit in early stroke outcome (8). Edaravone suppresses ROS, inhibits vascular endothelial injuries, and protects the neurovascular unit. No benefit was found for HT occurrence.

Neutrophils are one of the first innate immune cells to reach the ischemic brain tissue (129). Neutrophils contribute to injury through inflammatory mediators and damage the BBB through the release of proteolytic enzymes, ROS, and interactions with the endothelium of cerebral blood vessels. In addition, neutrophils accumulate in higher numbers in regions of HT and correlate with disruption of the basal lamina. In experimental stroke studies, depletion of circulating neutrophils reduces thrombolysis-related hemorrhage (130). In 846 patients treated with thrombolysis, an increased neutrophil to leukocyte (NIL) ratio was independently associated with HT (117).

Neutrophils produce neutrophil extracellular traps (NETs), which can promote clot formation associated with resistance to thrombolysis (131). In cultured human neutrophils, incubation with alteplase induced neutrophil degranulation and an increase in MMP-9 activity in a dose-dependent manner (132). This may elevate the number of activated neutrophils, thus exacerbating the detrimental effects of neutrophils on the BBB to promote HT (129, 131). In mice with circulating leukocytes from MMP-9 null bone marrow, BBB disruption was decreased (133).

Leukocytes enter the parenchyma and adhere to the cells largely through adhesion molecules ICAM-1, P-selectin, E-selectin, and VCAM-1 (134). Cytokines released by cerebral ischemia such as TNF, IL-1, and IFN-γ up-regulate ICAM-1 on both cerebral endothelial cells and leukocytes. Leukocytes adhering the endothelium can block erythrocytes in the microvasculature, leading to further injury (135). Activated leukocytes can also lead to secondary injury by producing proteases, MMPs, and ROS that can disrupt blood vessels. Leukocyte derived MMP-9s are mediators of early HT (1). Leukocyte adhesion and migration across the vasculature activates a number of signaling cascades (protein kinase C, focal adhesion kinase) that increase the BBB permeability. Once in the CNS, activated leukocytes release inflammatory cytokines at the site of injury such as TNF, IL-1β, and IL-6 (136).

In both rat (11) and rabbit models (12), treated with alteplase plus N-tert-butyl-α-phenylnitrone (PBN) vs. alteplase alone, PBN was found to attenuate alteplase-induced HT. PBN is a spin trap agent, targeting ROS from leukocytes.

## Inflammatory Genes and HT

In both patient and animal studies, the peripheral immune response in ischemic stroke can be characterized by RNA expression in circulating leukocytes (128). Using leukocyte RNA obtained before thrombolysis treatment in 44 patients with acute stroke (33 with HT), microarray gene expression studies have identified 6 genes that predict the occurrence of HT with 80% sensitivity and 70% specificity. The six-gene panel in Table 8 highlights differences in inflammation and coagulation processes. Factors involved in HT of human stroke include a shift in growth factor-beta signaling involving SMAD4, INPP5D, and IRAK3, a disruption of coagulation factors V and VIII, and amphiregulin, a growth factor-beta that regulates MMP-9. Additionally, increased expression of AKAP7 in peripheral lymphocytes is linked to post stroke complications and BBB permeability (137). Genes associated with increased HT and mortality risk include two single nucleotide polymorphisms rs669 in α-2-macroglobulin (A2M) and rs1801020 in coagulation Factor XII (138). Factors associated with the efficacy of alteplase include low fibrin levels and polymorphism of Factor XIII V/V genotype (137).

**Table 8:** Leukocyte genes associated with HT in patients with ischemic stroke.

**Gene**	**Function**	**Direction of expression**
SMAD4	Codes for a member of the Smad family of signal transduction proteins, which are activated by TGF-β signaling to regulate the of target genes	Increased
INPP5D	Regulates proliferation and programming of myeloid cell	Increased
VEGI	Codes for a cytokine in the tumor necrosis factor ligand family	Decreased
AREG	Codes for a ligand for the epidermal growth factor receptor	Increased
MCFD2	Involved in the transport of coagulation factors V and VIII from the endoplasmic reticulum to the Golgi apparatus	Decreased
MARCH7	Regulates membrane receptor expression in several tissues, including leukocytes	Increased

The Genot-PA score, to predict HT in patients treated with alteplase, considered both genetics and clinical variables to establish patient risk (137). In the trial, 1,324 patients in a Spanish population, were risk-stratified and treated with either alteplase or a combination of alteplase and mechanical thrombectomy. The study was able to predict HT in patients treated with alteplase. External validation of the score is pending.

## Conclusions

Inflammation contributes to BBB disruption and risk of HT, through an increase in ROS, MMPs and neutrophil activation. Clinical factors associated with inflammation and HT include hypertension, hyperglycemia, and age. Diabetes and hypertension lead to structural and functional changes of the neurovascular unit, alter collaterals, and elevate MMP-9, ROS, and proinflammatory cytokines (TNF, IL-1β, and IL-6). Advancing age is associated with a chronic elevation of inflammatory cytokine levels and changes in inflammatory response. These inflammatory markers provide insight to how clinical factors may increase the risk of HT. They may represent novel targets to reduce the risk of HT.

Further studies of the immune system and inflammation will be useful to better understand the risk of HT associated with reperfusion following thrombolysis and EVT and determine if modulation of the immune system could be useful to prevent HT. With increased use of reperfusion therapy and extension of eligibility criteria (e.g., inclusion of older patients, extended time windows), the importance of preventing HT will become more apparent. An improved safety profile with lower risk of HT could permit more widespread use of reperfusion therapy and greatly affect patients with stroke. Incorporating clinical features, genetics, biomarkers, and imaging to identify patients at risk for HT may help develop novel therapies to prevent HT in stroke.

## Author Contributions

All authors meet the authorship criteria and contributed substantially to conception and design or acquisition of data (ES and GJ), or analysis and interpretation of data (all authors), drafted (ES) or revised (all authors) and gave final approval of the version to be published (all authors).

## Conflict of Interest

The authors declare that the research was conducted in the absence of any commercial or financial relationships that could be construed as a potential conflict of interest.

## Abbreviations

A2M: a-2-macroglobulin
aICH: asymptomatic intracranial hemorrhage
AIS: acute ischemic stroke
Ang: angiopoietins
BBB: blood-brain barrier
c-Fn: cellular-fibronectin
DAMPs: damage associated molecular patterns
DM: diabetes mellitus
E-selectin: endothelial-leukocyte adhesion molecule 1
ECASS: European Cooperative Acute Stroke Study
eNOS: endothelial NO synthase
EVT: endovascular therapy
FGF: fibroblast growth factor
HgA1c: hemoglobin A1c
HI1: small petechial hemorrhagic infarction
HI2: confluent petechial hemorrhagic infarction
HIF-1: hypoxia-inducible factor 1
HMGB1: high mobility group box protein 1
HT: hemorrhagic transformation
ICAM-1: intercellular adhesion molecule-1
IFN: interferon
IGF-1: insulin-like growth factor 1
IL: interleukin
iNOS: inducible nitric oxide synthase
IV-tPA: intravenous tissue plasminogen activator
LPR: lipoprotein receptor
MCP-1: monocyte chemoattractant protein 1
MMP: matrix metalloproteinase
mRS: modified Rankin Scale
NETs: neutrophil extracellular traps
NFκB: nuclear factor-κB
NIHSS: National Institutes of Health Stroke Scale
NIL: neutrophil to leukocyte ratio
NLRP3: NOD-like receptor protein 3
NO: nitric oxide
NOX2: NADPH oxidase 2
NVU: neurovascular unit
NXY-059: disodium 2, 4-sulphophenyl-N-tert-butylnitrone
ONOO^−^: peroxynitrite
PAI-1: plasminogen activator inhibitor-1
PAR-1: protease activated receptor 1
PDGF-CC: platelet derived growth factor-CC
PDGFRα: platelet derived growth factor receptor alpha
PH1: small parenchymal hemorrhage
PH2: large parenchymal hemorrhage
RAGE: receptor for advanced glycation end products
ROS: reactive oxygen species
RNS: reactive nitrogen species
SBP: systolic blood pressure
SHR: spontaneously hypertensive rat
sICAM: soluble intercellular adhesion molecule
sICH: symptomatic intracranial hemorrhage
TAFI: thrombin-activated fibrinolysis inhibitor
TGF-β: transforming growth factor-beta
TJP: tight junction proteins
TLR: toll like receptor
TNF: tumor necrosis factor
TNK: Tenecteplase
tPa: tissue plasminogen activator
TXNIP: thioredoxin interacting protein
VAP-1: vascular adhesion molecule-1
VCAM-1: vascular cell adhesion molecule 1
VEGF: vascular endothelial growth factor.
